# Disruption of Atg7-dependent autophagy causes electromotility disturbances, outer hair cell loss, and deafness in mice

**DOI:** 10.1038/s41419-020-03110-8

**Published:** 2020-10-24

**Authors:** Han Zhou, Xiaoyun Qian, Nana Xu, Shasha Zhang, Guangjie Zhu, Yuan Zhang, Dingding Liu, Cheng Cheng, Xiaocheng Zhu, Yongze Liu, Ling Lu, Jie Tang, Renjie Chai, Xia Gao

**Affiliations:** 1 Department of Otolaryngology Head and Neck Surgery, Affiliated Drum Tower Hospital of Nanjing University Medical School, Jiangsu Provincial Key Medical Discipline (Laboratory), No. 321 Zhongshan Road, 210008 Nanjing, China; 2 Department of Physiology, School of Basic Medical Sciences, Southern Medical University, 510515 Guangzhou, China; 3 MOE Key Laboratory for Developmental Genes and Human Disease, School of Life Sciences and Technology, Jiangsu Province High-Tech Key Laboratory for Bio-Medical Research, Southeast University, 210096 Nanjing, China; 4 Children’s Hospital of Nanjing Medical University, 210008 Nanjing, China; 5 Guangdong-Hong Kong-Macao Greater Bay Area Center for Brain Science and Brain-Inspired Intelligence, Southern Medical University, Guangzhou, China; 6 Co-Innovation Center of Neuroregeneration, Nantong University, 226001 Nantong, China; 7 Institute for Stem Cell and Regeneration, Chinese Academy of Science, Beijing, China; 8 Research Institute of Otolaryngology, No. 321 Zhongshan Road, 210008 Nanjing, China; 9 Beijing Key Laboratory of Neural Regeneration and Repair, Capital Medical University, 100069 Beijing, China

**Keywords:** Macroautophagy, Cochlea

## Abstract

Atg7 is an indispensable factor that plays a role in canonical nonselective autophagy. Here we show that genetic ablation of *Atg7* in outer hair cells (OHCs) in mice caused stereocilium damage, somatic electromotility disturbances, and presynaptic ribbon degeneration over time, which led to the gradual wholesale loss of OHCs and subsequent early-onset profound hearing loss. Impaired autophagy disrupted OHC mitochondrial function and triggered the accumulation of dysfunctional mitochondria that would otherwise be eliminated in a timely manner. Atg7-independent autophagy/mitophagy processes could not compensate for Atg7 deficiency and failed to rescue the terminally differentiated, non-proliferating OHCs. Our results show that OHCs orchestrate intricate nonselective and selective autophagic/mitophagy pathways working in concert to maintain cellular homeostasis. Overall, our results demonstrate that Atg7-dependent autophagy plays a pivotal cytoprotective role in preserving OHCs and maintaining hearing function.

## Introduction

Macroautophagy (henceforth referred as autophagy) is conserved in all eukaryotes that degrades damaged proteins, nucleic acids, and cellular organelles. Autophagy can be induced in response to physiological/pathological stimuli and helps to maintain cytosolic homeostasis^1^. To date, >30 autophagy-related genes (Atgs) have been identified^2^. Mammalian Atgs can be subdivided into six functional clusters, including the Atg5/Atg12 system and Atg8/light chain 3 (LC3) system, and within both these systems Atg7 plays a fundamental role. Atg7 adenylates Atg12 and Atg8/LC3 and forms a thioester intermediate with both and ultimately transfers them to Atg3 and Atg10^3,4^.

In contrast to the embryonic lethality observed in Ambra1^−/−^, FIP200^−/−^, and beclin 1^−/−^ mouse phenotypes, Atg7^−/−^ mice, similar to Atg5^−/−^, Atg3^−/−^, Atg9^−/−^, and Atg16L1^−/−^ knockouts, die within the neonatal period^5^. To investigate the roles of autophagy in mammals, Atg7 and Atg5 conditional knockout mice were created by the Cre-Loxp method^6,7^. A line of transgenic mice expressing the Cre recombinase were bred with Atg7^flox/flox^ or Atg5^flox/flox^ mice, and most of the homozygous mice exhibited pathological phenotypes^5,8^.

Cochlear hair cells (HCs) are postmitotic cells that rely on cellular homeostasis to maintain cell survival, but the role of autophagy in keeping cellular homeostasis in the mammalian inner ear is still poorly understood. Initial work on cochlear autophagy dates back 40 years to when Hinojosa observed autophagic vacuoles containing cell organelles in maturing cochlear cells during postnatal development and remodeling^9^. It was not until 2007, however, that this field sparked more intense research interest. Taylor et al. described the pattern of cochlear HC lesions in postnatal day (P) 18–P21 mice induced by coadmininstration of kanamycin and bumetanide^10^. Large numbers of outer hair cells (OHCs) died via an apoptotic pathway, but different modes of subsequent inner hair cell (IHC) death were indicated, including autophagy. In 2015, de Iriarte Rodríguez et al. analyzed the cochlear transcriptome of the mice^11^. At P270, as compared to embryonic day 18.5, LC3-II was increased while p62 was decreased, which indicated that the expression of autophagy-related genes is regulated throughout cochlear maturation and aging. Also in 2015, Yuan et al. studied autophagy in P90 adult mice subjected to a 2–20 kHz broadband 96/106 dB noise model and observed a temporary threshold shift at 96 dB and a permanent threshold shift at 106 dB. The LC3B level was elevated after 96 dB (but not 106 dB) noise exposure and promoted OHC survival^12^. The landmark study involving inner ear HC-specific autophagic gene conditional knockout mice was reported by Fujimoto et al. in 2017^13^. In their work, Pou4f3^Cre^Atg5^flox/flox^ mice were generated by deletion of Atg5 specifically in HCs. The mutant mice had profound hearing loss. At P14, many stereocilia were destroyed in OHCs and IHCs, and the cell bodies of some OHCs were damaged, and at P60 nearly all OHCs were missing. However, the reason why OHCs were more vulnerable to deficient autophagy was not determined in their work.

In this study, we targeted OHC autophagy by taking advantage of OHC-specific *Atg7* conditional knockout mice. We found that disruption of Atg7-dependent autophagy in OHCs resulted in stereocilium damage, somatic electromotility disturbances, and presynaptic ribbon degeneration, which eventually led to OHC loss and hearing impairment in mice. These results suggest that Atg7-dependent autophagy protects the health of OHCs, thus maintaining hearing function.

## Materials and methods

### Animals

Atg7^flox/+^ mice (Stock #D000534) and Prestin^Cre/+^ mice (Stock #D000023) of both sexes were bought from Model Animal Research Center of Nanjing University and used in the experiments. Atg7^flox/+^ mice were bred with Atg7^flox/+^ mice to get Atg7^flox/flox^ mice, and Atg7^flox/+^ mice were bred with Prestin^Cre/+^mice to get Prestin^Cre/+^Atg7^flox/+^ mice. Atg7^flox/flox^ mice were then crossed with Prestin^Cre/+^Atg7^flox/+^ mice to produce Prestin^Cre/+^Atg7^flox/flox^ mice (with Atg7^flox/flox^ mice as controls). Most of the experiments were done using mice at P30, P60, and P90. Mice were housed in pathogen-free facilities with controlled day–night cycles. All animal protocols were approved by the Ethics Review Committee of Nanjing University and were in accordance with the National Institute of Health’s Guide for the Care and Use of Laboratory Animals.

### Genotyping PCR

Genomic DNA from toes or tail tips were genotyped by adding 50 mM NaOH, incubating at 98 °C, and neutralizing with 1 M Tris-HCl (Solarbio, T1130). Primers were as follows: Atg7: (F) 5’-TGG CTG CTA CTT CTG CAA TGA TGC-3’; (R) 5’-CAG GAC AGA GAC CAT CAG CTC CAC-3’; wild type: (F) 5’-ATT GTG GCT CCT GCC CCA GT-3’; (R) 5’-CAG GAC AGA GAC CAT CAG CTC CAC-3’; Prestin: (F) 5’-ATT TGC CTG CAT TAC CGG TC-3’; (R) 5’-ATC AAC GTT TTC TTT TCG G-3’. PCR cycles were an initial denaturing step of 3 min at 94 °C, 38 cycles of 30 s denaturation at 94 °C, 30 s annealing at 58 °C, 30 s extension at 72 °C, and cooling at 4 °C.

### Histological examination

Isolated cochleae were immersed in 4% paraformaldehyde and then decalcified with 0.5 M EDTA (Solarbio, E1170). For cryosectioning, cochleae were immersed in increasing concentrations of 10–30% (w/v) sucrose (Biosharp, Amresco 0335) and then with serial mixtures of OCT (Sakura, Tissue-Tek 4583) and sucrose. The sections and whole mounts were blocked with phosphate-buffered saline blocking solution containing 5% donkey serum, 1% bovine serum albumin, 0.02% sodium azide (Sigma-Aldrich, S8032), and 0.5% Triton; incubated with diluted primary antibodies; and further with fluorescence-conjugated secondary antibodies (Alexa Fluor 488/555/647, Invitrogen). The primary antibodies were Atg7 rabbit polyclonal antibody (Thermo Fisher, PA5-35203, 1:300); Myosin VIIa mouse polyclonal antibody (Thermo Fisher, PA1-936, 1:500); Prestin goat polyclonal antibody (Santa Cruz, sc-22694, 1:200); CtBP2 mouse monoclonal antibody (BD Biosciences, 612044, 1:200); and P62 Guinea Pig polyclonal antibody (Progen, GP62-C, 1:200). The anti-fade Fluoromount-G mounting medium (SouthernBiotech, 0100-01) was used for mounting. The fluorescence images were obtained by a Zeiss LSM 710 confocal microscope. For hematoxylin staining, the whole mounts were stained with diluted hematoxylin (Solarbio, G1080) for 5 min.

### Reverse transcription PCR (RT-PCR)

RNA of the cochlea and other organs was extracted using the Total RNA Extractor (Sangon Biotech, B511311) with a pellet pestle motor (DGS, G55500), transcribed by the ReverseAid First Strand cDNA Synthesis Kit (Thermo Fisher, K1622), and underwent RT-PCR. The concentration and purity were identified by NanoDrop (Thermo Fisher, 2000). The primers were: Atg7: (F) 5’- ATG CCA GGA CAC CCT GTG AAC TTC-3’; (R) 5’- ACA TCA TTG CAG AAG TAG CAG CCA-3’; β-actin: (F) 5’- ACG GCC AGG TCA TCA CTA TTG-3’; (R) 5’-AGG GGC CGG ACT CAT CGT A-3’.

### Western blot

Cochleae and other organs were dissected and transferred to the homogenizer tubes and mixed with RIPA lysis buffer (Fudebio, FD008) with a protease inhibitor cocktail (Roche, 11697498001). The supernatant from centrifuged homogenates was mixed with sodium dodecyl sulfate buffer (Beyotime, P0015L), boiled, electrophoresed, and blotted onto a 0.2-μm polyvinylidene difluoride membrane (Millipore, Immobilon ISEQ00010). The primary antibodies were: Atg7 rabbit polyclonal antibody (Thermo Fisher, PA5-35203, 1:500), Prestin goat polyclonal antibody (Santa Cruz, sc-22694, 1:400), GAPDH mouse monoclonal antibody (Abcam, ab9484, 1:1000), β-Actin rabbit IgG antibody (Abmart, P30002, 1:1000), LC3 rabbit polyclonal antibody (CST, 4108, 1:1000), and P62 Guinea Pig polyclonal antibody C-terminal specific (Progen, GP62-C, 1:500). The ECL Kit and horseradish peroxidase-conjugated antibodies were used for detection. Images were obtained by GE ImageQuant LAS4000.

### Auditory brainstem response (ABR) and distortion product otoacoustic emissions (DPOAEs)

Mice were intraperitoneally anesthetized by 0.01 g/ml pentobarbital sodium (100 mg/kg). A TDT workstation (Tucker-Davis Technologies, System3) running SigGen32 software was used for ABR and DPOAE tests in a soundproof room. Broadband clicks and 4, 8, 16, 24, and 32 kHz tone pips were generated. The open-field ABR waveforms were recorded with needle electrodes at the vertex (active), the posterior bulla region of the ear (reference), and the nasal tip (ground). Auditory thresholds were identified by decreasing the sound intensity and distinguishing the ABR wave I. DPOAE testing was equipped with an ER-10B (Etymotic Research) and a probe. DPOAE was evoked by two simultaneously applied long-lasting constant-level pure tones (L1 = 65 dB SPL and L2 = 75 dB SPL) with a frequency ratio (F2/F1) of 1.2 given through earphones. DPOAE was recorded at 2F1 − F2 frequency and plotted as a function of the F2 frequency within the range of 1–32 kHz. DPOAE was considered present when at least it was 3 dB above the average noise floor.

### Scanning electron microscopy (SEM) and transmission electron microscopy (TEM)

Isolated cochleae were immersed in 2.5% glutaraldehyde (Alfa Aesar, A17876). Cochlear whole mounts were fixed in 1% OsO_4_, dehydrated through graded ethanol, and desiccated by a CO_2_ critical-point dryer (Leica, EM CPD300). For SEM, samples were sputter-coated with gold (Cressington, 108) on stubs. A field-emission scanning electron microscope (FEI, Quanta250) was used to obtain images. For TEM, samples were further infiltrated in a graded series of propylene oxide (Macklin Biochemical, P816084) and gradually polymerized in araldite. Ultrathin sections were made by a Leica EM UC6 powertone, sequentially post-stained with uranyl acetate and lead citrate, and examined by a Hitachi H-7650 transmission electron microscope.

### Electrophysiology

Under a dissection microscope, the bony otic capsule of the isolated cochleae was removed using fine forceps in a 35-mm Petri dish with 3 ml Leibovitz L-15 medium (Thermo Fisher, 11415064) buffered with 10 mM HEPES to an osmolality of 300 mOsm (Gonotec, Osmomat 3000). After removing the stria vascularis, the whole mount was released from the modiolus, cut into three pieces, and transferred to 100 μl L-15 medium with diluted collagenase IV (Sigma, C5138) for 5 min. The enzymatic digestion solution was then replaced by enzyme-free L-15 medium. The OHCs of the middle turn were gently swept out using a superfine eyelash with a handle (Ted Pella, 113) and isolated. Borosilicate glass filaments (Sutter instruments, B150-86-10) were pulled by a Sutter P-2000 and then polished with a microforger (Narishige, MF380) to make 2–3 μm patch electrodes with an initial resistance around 3 MΩ. The OHC cell membrane nonlinear capacitance (NLC) was measured using a continuous two sine wave stimulus protocol superimposed onto a voltage ramp from −120 to +100 mV. The low-pass filtered currents were amplified by an Axopatch 200B (Axon Instruments). The pClamp 10 software on a computer connected to a Digidata 1440A A/D converter (Axon Instruments) was used to acquire whole-cell currents and evoked responses. The data were obtained using the jClamp 32 software.

### Statistical analysis

At least three independent experiments were performed for each experimental condition. Data were shown as mean ± S.E.M. Data were analyzed by the GraphPad Prism 6.02 software and two-tailed, unpaired Student’s *t* tests were used. Statistical results were labeled with */**/*** for *p* < 0.05, *p* < 0.01, and *p* < 0.001, respectively. Sample sizes were pre-determined by calculations derived from our experience. Animals were not randomly assigned during collection, but the data analysis was single masked. No sample was excluded from the analyses. The number of replicates was indicated in each figure legend.

## Results

### Atg7 is expressed in OHCs and IHCs

**Fig. 1:**
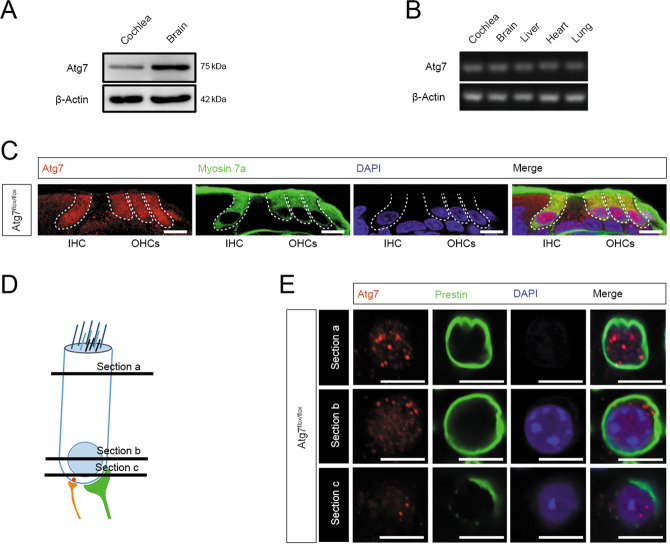
OHCs and IHCs expressed the highest levels of Atg7 in the cochlea. **A** Representative immunoblot of Atg7 protein in the cochlea. **B** Expression of the *Atg7* gene was analyzed by RT-PCR. **C** Atg7 was evident in OHCs and IHCs in the cochlear cryosections. **D**, **E** Atg7 puncta were distributed throughout the OHC cell bodies as indicated in the infracuticular, nuclear, and basal pool sections. *N* = 6 for each group (western blot: *N* = 3). Scale bar: **A**–**C**: 5 μm; **D**, **E**: 10 μm.

**Fig. 2:**
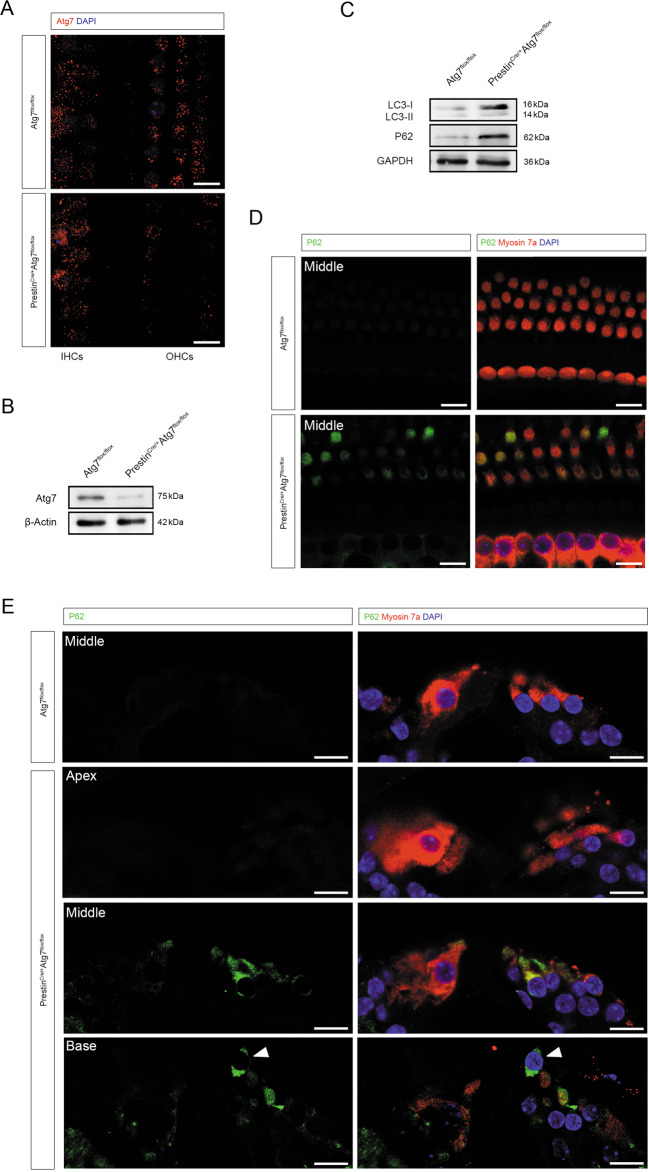
Abnormal autophagy in Atg7-deficient OHCs. **A** Representative immunostaining images showing efficient knockout of Atg7 in the OHCs and not in the IHCs. **B** Western blot showed that Atg7 was largely suppressed in the cochlear whole mount of Prestin^Cre/+^Atg7^flox/flox^ mice. **C** Representative immunoblots showing elevated P62, increased LC3-I, and decreased LC3-II/LC3-I ratio. **D** P62 accumulated in Atg7-deficient OHCs, showing a colorful mosaic pattern when co-stained with myosin 7a. **E** P62 aggregated in the mutant OHCs, especially at middle and basal turns. An OHC in the basal turn was extruded from the sensory epithelium (white arrowhead). *N* = 6 for each group (western blot: *N* = 3). Scale bar: 10 μm.

Western blot demonstrated that Atg7 was expressed in the cochlea and brain (Fig. 1A), and RT-PCR confirmed *Atg7* mRNA expression in the cochlea (Fig. 1B). Immunostaining of cryosections showed that HCs had the highest expression level of Atg7 compared with supporting cells and other surrounding cells in the organ of Corti (Fig. 1C), and whole-mount immunostaining showed Atg7 puncta in the cell bodies of OHCs and IHCs (Fig. 1D, E, 2A).

### Generation of OHC-specific Atg7 conditional knockout mice

Atg7 was inactivated in OHCs by crossbreeding the Atg7^flox/flox^ mice with Prestin^Cre/+^Atg7^flox/+^ mice to obtain the Prestin^Cre/+^Atg7^flox/flox^ mouse strain, and the Atg7^flox/flox^ mice without Cre were used as controls. PCR amplification of fragments derived from wild-type and mutant alleles showed bands of 460 and 550 bp for Atg7 and 350 bp for Prestin^Cre/+^. OHC-specific Atg7-deficient mice were born at Mendelian frequency, healthy and fertile, and did not show any overt phenotype compared to their Atg7^flox/flox^ siblings.

### Aberrant autophagy in OHCs upon Atg7 deficiency

The Atg7 knockout efficiency was determined by immunostaining and western blotting and compared with the control OHCs from Atg7^flox/flox^ mice at P30. Atg7 was largely suppressed in the OHCs of Prestin^Cre/+^Atg7^flox/flox^ mice (Fig. 2A). Atg7 protein expression level in the cochlear whole mount (without spiral ganglion neurons) was dramatically reduced (Fig. 2B). Immunoblots of LC3 and P62 (SQSTM1) showed increased LC3-I, decreased LC3-II/LC3-I ratio, and elevated P62, supporting autophagy deficiency in our model (Fig. 2C). Furthermore, immunostaining of the cochlear whole mount revealed that P62 accumulated in Atg7-deficient OHCs as early as P30 (Fig. 2D). In the cryosectioned samples at P30, we demonstrated that P62 aggregated in the cell bodies of the mutant OHCs, especially at middle and basal turns (Fig. 2E).

### Prestin^Cre/+^Atg7^flox/flox^ mice have an abnormal morphological phenotype

**Fig. 3:**
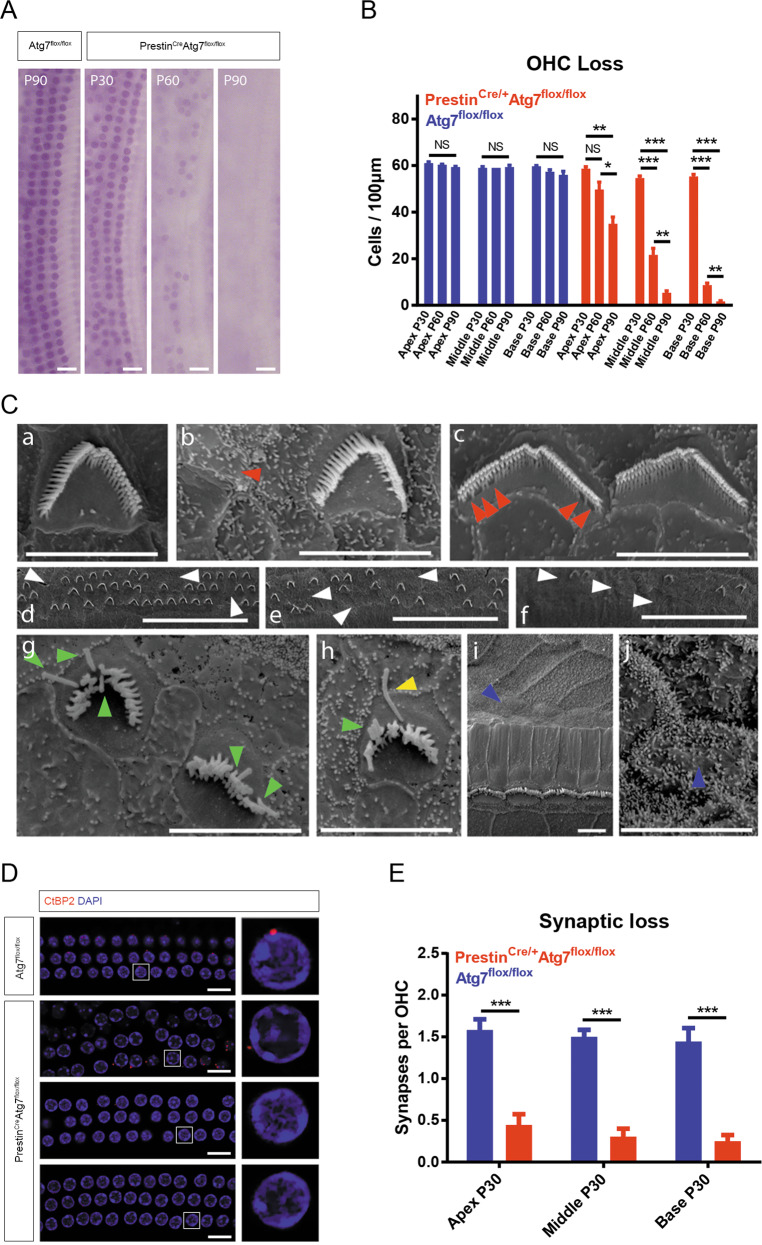
Morphological changes in Prestin^Cre/+^Atg7^flox/flox^ mice and controls. **A** Representative hematoxylin staining images of the cochlear middle turn depicting the gradual loss of OHCs over time. **B** The average number of OHCs per 100 μm dropped by P30 and dramatically decreased at P60 and P90, especially in the basal turns. **C** SEM images from control mice (a) and Atg7-knockout mice (h–j). (b) A bundleless OHC (red arrowhead). (c) Stereocilia were missing from the inner row (red arrowheads), while the regular V/W-shaped staircase structure was maintained. (d–f) Three consecutive fields of an apical turn, within which part c was closer to the middle turn and part a was more apical. OHCs of all three rows randomly lost their hair bundles (white arrowheads). (g, h) At P60, the stereocilia were degenerated. Splayed stereocilia and persistent kinocilia were identified (green and yellow arrowheads, respectively). (i, j) The dead OHCs were replaced by supporting cells with microvilli (blue arrowheads). **D**, **E** Immunolabeling of CtBP2 (red channel) showed the loss of presynaptic ribbons of OHCs. Insets to the right represent typical OHCs with or without ribbons. *Z*-stack quantification of CtBP2 puncta per OHC showed significant differences between Atg7 knockouts and controls. *N* = 6 for each group. Scale bar: **A**: 20 μm; **C**(a–c, g–j): 5 μm; **C**(d–f): 50 μm; **D**: 10 μm.

Hematoxylin staining of cochlear whole mounts showed that Prestin^Cre/+^Atg7^flox/flox^ mice had occasional OHC loss at P30. OHCs were lost at an accelerated rate thereafter, with a gradient from the basal turn toward apical. At P60, the basal-turn OHCs were largely lost, while over half of the middle-turn OHCs also disappeared. At P90, all OHCs in the basal and middle turns were lost and just a few apical OHCs remained. However, in stark comparison, Atg7^flox/flox^ mice had almost no OHC loss even at P90 (Fig. 3A, B). SEM showed a similar trend of base-to-apex OHC stereocilium loss in Prestin^Cre/+^Atg7^flox/flox^ mice (Fig. 3C). The stereocilium of the OHCs was progressively degenerated from P30 to P60. At P30, except for a few rare cells, Atg7-knockout OHCs presented a regular “V” or “W”-shaped staircase structure. At P60, however, the stereocilia became splayed or fused and portions of the hair bundles were lost (Fig. 3Cg). Some OHCs had completely pinched off their damaged hair bundles along with a bit of apical cytoplasm, resulting in a bundleless HC. Surprisingly, several surviving OHCs still possessed a single kinocilium (Fig. 3Ch). At P90, the OHCs exhibited extensive extrusion from the epithelium. In contrast, the stereocilium of OHCs from the control groups remained normal. There was also a significant loss of presynaptic ribbons in Atg7-deficient OHCs throughout the cochlea at P30. OHC afferent synapses were rendered visible by immunostaining for a component of the presynaptic CtBP2 protein. The average numbers of CtBP2 puncta per mutant OHC in the apical/middle/basal turns were 0.44 ± 0.13/0.31 ± 0.10/0.25 ± 0.07 versus 1.58 ± 0.13/1.50 ± 0.08/1.44 ± 0.16 in control OHCs, respectively, with all *p* values <0.001. Furthermore, OHC synaptic ribbons in the apical turn seemed more resistant to Atg7 deficiency than OHCs in the middle and basal turns (Fig. 3D, E). All of these forms of damage to the delicate OHC structures resulted in permanent loss of function.

### Prestin^Cre/+^Atg7^flox/flox^ mice have impaired hearing function

**Fig. 4:**
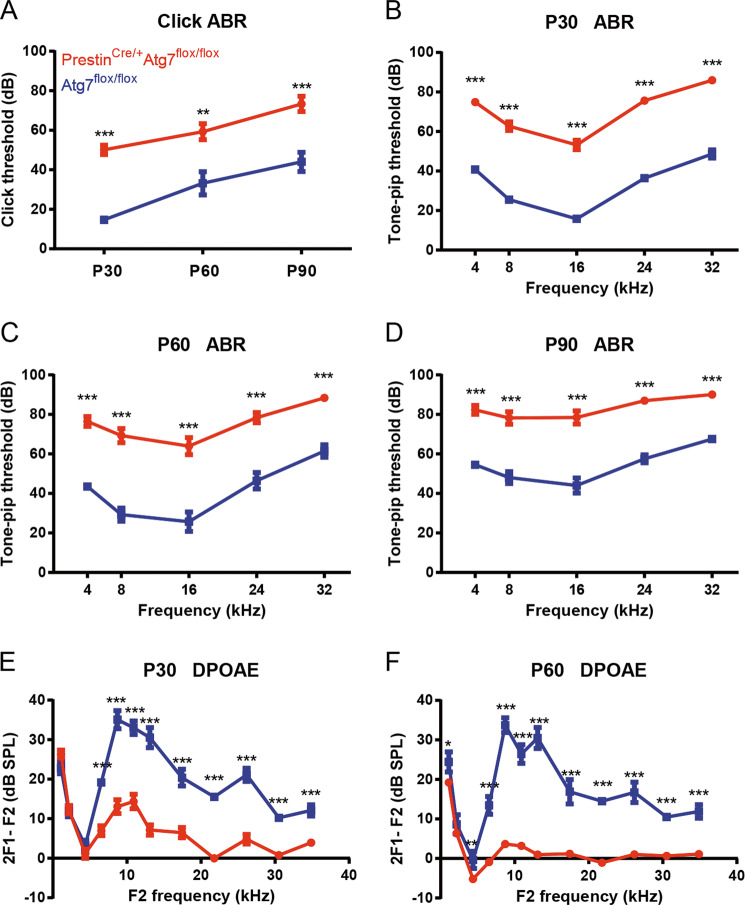
Severe hearing loss in Prestin^Cre/+^Atg7^flox/flox^ mice. **A** ABR click thresholds showed a 40-dB gap between two mice groups. **B** At P30, the hearing thresholds of Prestin^Cre/+^Atg7^flox/flox^ mice were >70 dB on average. **C** At P60, the average ABR hearing thresholds of Prestin^Cre/+^Atg7^flox/flox^ mice were elevated to >75 dB. **D** At P90, the ABR hearing thresholds were around 83 dB for the knockout mice. **E**, **F** DPOAE traces with 2F1 − F2 measured as a function of frequency F2. The strongest response was around 10 kHz at the F2 frequency for Prestin^Cre/+^Atg7^flox/flox^ mice. At P30, the DPOAE of the Atg7-deficient mice was suppressed at frequencies >6 kHz, with an overall average shift of >11 dB. At P60, the difference between the two groups approached 15 dB on average. DPOAE was almost absent at around ≥12 kHz. *N* = 12 for each group.

ABR click thresholds at P30, P60, and P90 showed that Prestin^Cre/+^Atg7^flox/flox^ mice suffered from accelerated hearing loss, and the control group had better hearing by >40 dB as early as P30 (Fig. 4A). ABR tone pip thresholds were measured at 4, 8, 16, 24, and 32 kHz. At P30, Prestin^Cre/+^Atg7^flox/flox^ mice lost their Preyer’s reflex, and their hearing thresholds across all frequencies were elevated by about 37 dB on average compared to the control group (Fig. 4B). At P60, the response thresholds of the Prestin^Cre/+^Atg7^flox/flox^ mice continued to increase, especially at 8 and 16 kHz, and the gap was about 34 dB on average (Fig. 4C). By P90, the Prestin^Cre/+^Atg7^flox/flox^ mice had developed severe hearing loss (Fig. 4D), and the hearing impairment seemed to propagate from high frequencies to low frequencies. DPOAE outputs were also assessed. At P30, DPOAEs were attenuated with closely packed peaks and troughs, and there was a significant difference from 6 to 32 kHz (Fig. 4E). At P60, Prestin^Cre/+^Atg7^flox/flox^ mice lacked detectable emissions at nearly all frequencies. Emissions at lower frequencies such as 4 kHz also showed statistically significant divergence compared with controls, which implied more apical involvement (Fig. 4F). Together, the ABR and DPOAE results suggested that all Atg7-deficient OHCs along the tonotopic map were affected; that mutant OHCs of higher frequencies were more vulnerable than those of middle and low frequencies; that at P30, despite generally normal morphology, many mutant OHCs had already lost most of their function; and that by P90 the majority of OHCs were degenerated and dead. These stark differences strongly suggest that Atg7 is required for OHC function and survival.

### Atg7 deficiency disrupts OHC electromotility

**Fig. 5:**
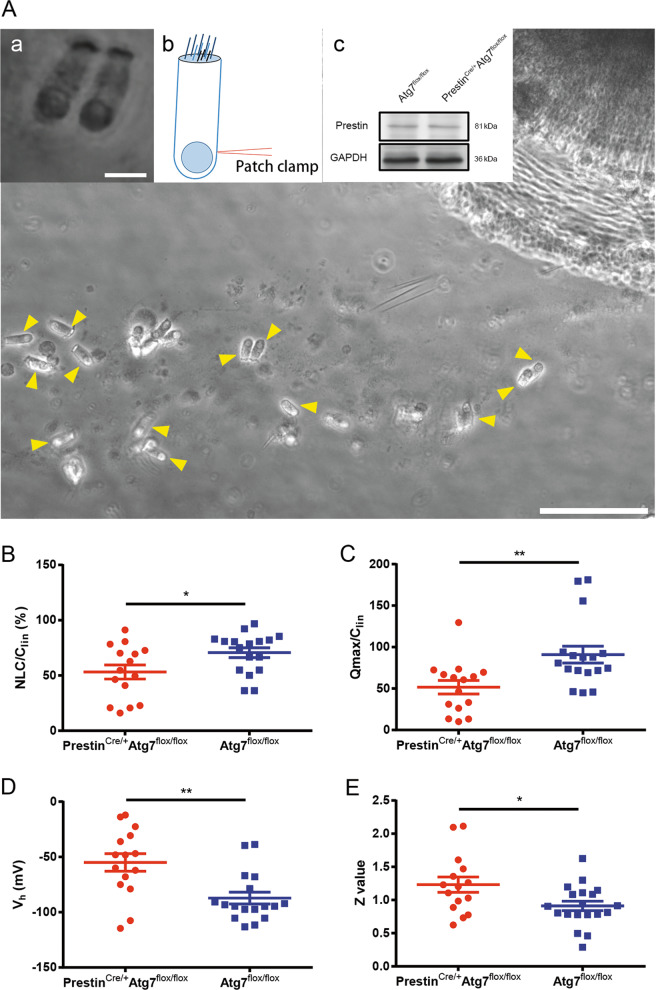
OHC electromotility was disturbed in Prestin^Cre/+^Atg7^flox/flox^ mice. **A** OHCs were dissected from the whole-mount cochleae, and HCs were indicated by yellow arrowheads. OHCs were differentiated from IHCs based on their characteristic morphology and unique electromotility, and two of them are shown in a. b shows the position where the electrode was patched onto the OHC body. Western blot showed the prestin expression in Prestin^Cre/+^Atg7^flox/flox^ mice at P30 (c). **B**–**E** NLCs recorded were pooled and normalized to the corresponding *C*_lin_. *Q*_max_, *C*_lin_, *V*_h_, and *z* were obtained from a curve fit of the NLC response for each OHC, as shown by scatter plots of individual data and normalized mean values with standard errors. Significant differences for these parameters were seen between the two groups. Atg7-mutant OHCs: *N* = 15; the control OHCs: *N* = 17 (western blot: *N* = 3 for each group). Scale bar: **A**: 100 μm; a: 10 μm.

OHCs are slender cylindrical cells with a cuticular surface, a rounded base, and a nucleus close to the lower end. We patched onto the OHC basolateral wall under the whole-cell patch-clamp configuration (Fig. 5A(a, b)) and recorded the voltage-dependent NLC, which is routinely used as a surrogate for electromotility. NLC traces of Prestin^Cre/+^Atg7^flox/flox^ and Atg7^flox/flox^ mice were recorded. The NLC capacitance data were fit to the first derivative of a two-state Boltzmann function as *C*_m_ = *Q*_max_*ɑ*/{exp[*ɑ*($\mathcal{V}$_m_ – $\mathcal {V}$_h_)](1 + exp[−*ɑ*($\mathcal {V}$_m_ – $\mathcal {V}$_h_)])^2^} + *C*_lin_, where *C*_m_ is the NLC, *C*_lin_ is the linear capacitance representing the membrane surface area, *Q*_max_ is the maximum voltage sensor charge moving through the membrane electric field and reflects the number of voltage-sensing proteins, *V*_h_ is the voltage at peak capacitance where the charge is distributed equally across the membrane, and *ɑ* is the slope factor describing the steepness of the voltage dependence (*ɑ* = *ze*/*kT*, where *z* is the apparent unitary charge movement or valence, *e* is the electron charge, *k* is the Boltzmann constant, and *T* is the absolute temperature). *V*_h_ and *z* define the voltage-operating range of the motor^14^.

NLC and *Q*_max_ were normalized to *C*_lin_ so as to be proportional to the varying plasma membrane area of OHCs. Four parameters (NLC/*C*_lin_, *V*_h_, *Q*_max_/*C*_lin_, and *z*) were analyzed. NLC/*C*_lin_ showed statistically significant differences (53.15 ± 6.35% versus 70.67 ± 4.72%, *p* = 0.0283) between the two groups. Also, we found that the NLC/*C*_lin_ of the Atg7-deficient OHCs exhibited a greater dynamic range than the control OHCs. Seven out of the 15 isolated Atg7-deficient OHCs had below average NLC/*C*_lin_ values, with the lowest being only 16.10%, while 10 out of the 17 control OHCs had NLC/*C*_lin_ values above the average and 12 of them had values close to the average (Fig. 5B). These results indicated that during voltage stimulation fewer prestin-associated gating charges were translocated. The *V*_h_ of the Atg7-deficient OHCs shifted toward more positive potentials compared to controls (−54.94 ± 7.93 versus −87.15 ± 5.72, *p* = 0.0018; Fig. 5C). A significant difference was found for *Q*_max_/*C*_lin_ between the two groups (51.61 ± 8.17 versus 90.97 ± 10.84, *p* = 0.0059; Fig. 5D). Interestingly, six OHCs had surprisingly low *Q*_max_/*C*_lin_ values (10.11 was the lowest), and a statistically significant divergence in the *z*-value of the two groups was seen (1.23 ± 0.12 versus 0.95 ± 0.07, *p* = 0.0194; Fig. 5E). Ten out of 15 Atg7-deficient OHCs had a *z*-value >1, including two OHCs whose value shifted to >2, which indicated the abnormally high voltage sensitivity of prestin. Although prestin expression was not changed in the knockout OHCs (Fig. 5Ac), the function of prestin was possibly changed due to autophagy ablation, which contributed to hearing impairment at P30.

### Loss of Atg7 in OHCs results in the accumulation of damaged mitochondria

**Fig. 6:**
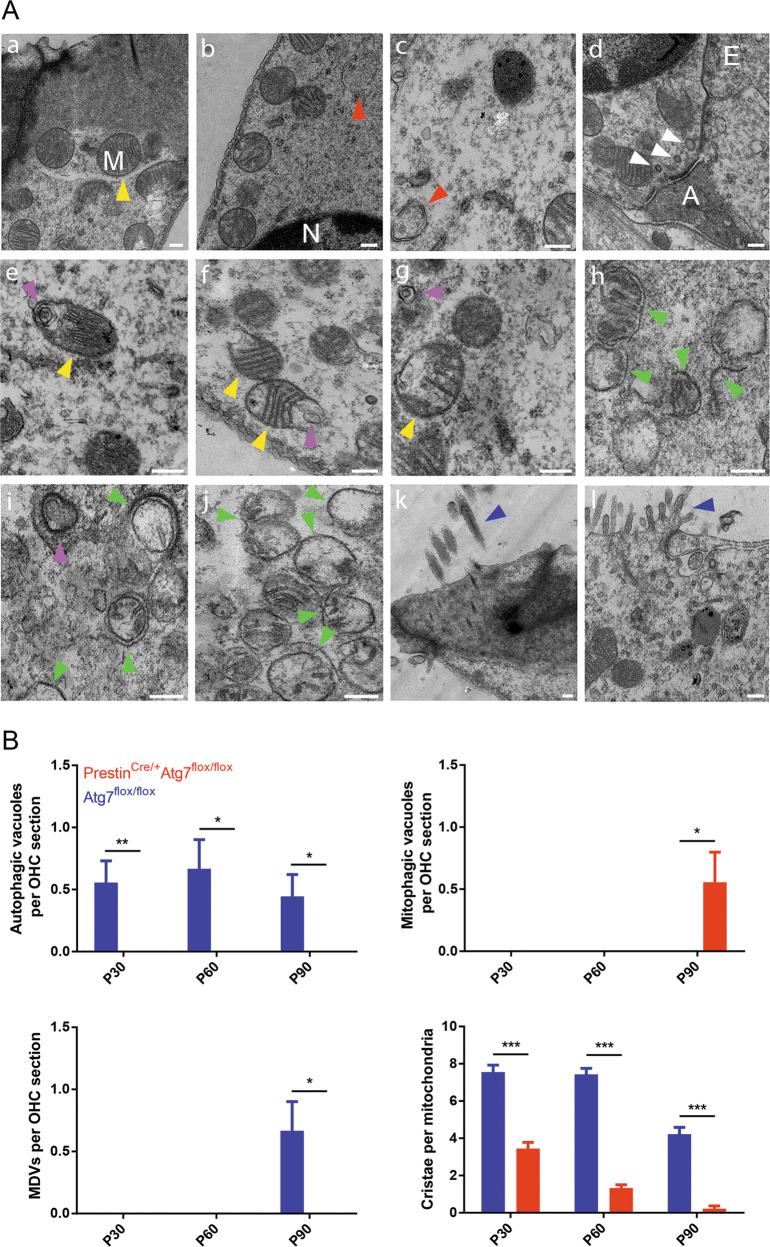
TEM micrographs of Atg7-deficient and control OHCs. **A** (a) A control OHC at P30. An infracuticular mitochondrion with lamellar cristae (yellow arrowhead). The outer and inner membrane could not be distinguished under low magnification. (b, c) A control OHC at P60 and another at P90, respectively, showing a double-membrane bulk nonselective autophagic vacuole containing cytoplasm (red arrowhead). (d) A control OHC at P30. Three ribbons of different sizes (white arrowheads) were located against the afferent synapse. A afferent synapse, E efferent synapse. (e–g) A control OHC at P90. (e) An MDV formed inside an OHC (pink arrowhead); (f) two “membrane-broken” mitochondria (yellow arrowheads). (g) An expelled MDV (pink arrowhead) and a nearby “membrane-recovered” mitochondrion (yellow arrowhead). (h–j) A representative Atg7-knockout OHC with abnormal mitochondria. (h) Sequestering membranes partially encapsulated degenerated mitochondria. Note that there was a sequestering membrane with atypical reverse-bending ends (green arrowheads). The outer membrane of the mitochondria became shriveled and blurred. (i) A mitophagosome indicated the existence of Atg7-independent mitophagy. (j) Cristae largely disappeared, and the remaining cristae became disorganized. The aberrant mitochondria were no longer constrained to their preferred locations. (k) A control OHC at P90 with normal stereocilia (blue arrowhead). (l) A degenerated mutant OHC was replaced by adjacent supporting cells with microvilli (blue arrowhead). **B** Number of autophagic vacuoles/mitophagic vacuoles/MDVs per OHC section and cristae per mitochondria between the two groups had statistical differences. *N* = 5 for each group. Scale bar: **A**: 200 nm.

OHC mitochondria are located in anatomically different regions, including the infracuticular and infranuclear regions and along the subsurface cisterns parallel to the lateral membrane where the metabolic demands require adequate energy turnover. The inner mitochondrial membrane locates the respiratory chain and ATP synthase complex^15^. We used TEM, the gold standard for gauging autophagy/mitophagy involvement, to corroborate OHC functional status. A bulk nonselective autophagic vacuole containing cytoplasm (but not organelles) was seen in the control OHCs but not in the knockout OHCs (Fig. 6A(b, c)), possibly due to the basal levels of autophagy. Control OHCs were richly endowed with mitochondria, and the architecture of the cristae could easily be deciphered. Mitochondria at various stages demonstrated their dynamic nature, necessitating a constant flux between degradation and replenishment (Fig. 6Aa). We were surprised to find mitochondria-derived vesicle (MDV) pathway involvement in control OHCs at P90 (Fig. 6A(e–g)), which had never been reported before. Strikingly, in OHCs of Prestin^Cre/+^Atg7^flox/flox^ mice, the dismantling and degradation process seemed to start from the inner membrane instead of the outer membrane of defective mitochondria. From as early as P30, protruding mitochondrial cristae of mutant OHCs started to shorten, swell, deform, collapse, or disappear (Fig. 6A(h–j)), which indicated reduced inner membrane surface area together with low-density mitochondrial mass, thus suggesting mitochondrial malfunction^16^. OHC destruction proceeded more quickly at the high-frequency end, and the accumulation of these damaged mitochondria triggered self-repair via mitophagy in order to remove them. During mitophagy, entire mitochondria are sequestered and engulfed in mitophagosomes and delivered to lysosomes for degradation^17^. We found degenerated mitochondria enclosed within mitophagosomes (Fig. 6Ah) but no elongated subtypes that could be spared from autophagy/mitophagy. Also, intrinsic mitochondrial fusion and fission were not observed. Furthermore, the vast majority of the mitochondria were only partially enwrapped by the limiting membranes, which implied that the Atg7-dependent autophagy/mitophagy machinery was disrupted during the membrane elongation process and thus was inadequate for selectively eliminating damaged mitochondria. Statistically significant differences in autophagic/mitophagic vacuoles, MDVs, and cristae per OHC section were seen (Fig. 6B). This degeneration process was different from OHC apoptosis, which is characterized by cell shrinkage, membrane blebbing, karyorrhexis, and apoptosome formation^18^. Massive mitochondria degeneration led to OHC degeneration, and the OHC corpses decomposed within the epithelium and the cellular debris was cleared (Fig. 6Al). Without coordinated mitochondrial biogenesis, a healthy respiring mitochondrial population inside the OHCs could no longer be maintained, resulting in the degeneration and ultimate demise of the OHCs.

## Discussion

### Atg7 is essential for the physiological function of OHCs

Properly functioning OHCs, together with IHCs, assure high sensitivity, sufficient dynamic range, and fine-grained frequency tuning^19,20^. OHCs are especially endowed with three major transduction systems: (1) mechanoelectrical transduction in the hair bundles, (2) electromechanical transduction in the lateral wall, and (3) electrochemical transduction at the synapses in the cell base.

OHC hair bundles are coordinated arrays of stereocilia containing mechanoelectrical transducer (MET) channels. Around P7, the MET current in cochlear HCs reaches its maximum amplitude, with rapid activation kinetics and current adaptation along the entire cochlea. In a previous study, uptake of the fluorescent dye FM1-43 into autophagy-deficient OHCs was not affected at P5, suggesting that Atg5-knockout OHCs have functioning MET channels at least until P5^13^. In the present study, Atg7-knockout OHCs occasionally lost some of their stereocilia at P30 as revealed by SEM, but considerable numbers of tip links and MET channels were still functional because DPOAE was not fully inhibited. This was consistent with a previous report that, at high SPL levels, the otherwise vulnerable DPOAE might be maintained due to the mechanical nonlinearity associated with stereociliary transduction (but not electromotility)^21^. However, at P60 the OHC stereocilia were aberrantly arranged or degenerated, with almost fully depressed DPOAE. The V/W-shaped staircase structure and planar cell polarization of the stereociliary bundles on top of each OHC depend on the oriented migration of the kinocilium. The kinocilium then regresses postnatally prior to the onset of hearing function at P12^22^. We found several surviving OHCs in the middle turn still bearing a single kinocilium at P60, which should be absent in adult mice. Taken together, our results in Atg7-deficienct OHCs suggest that autophagy is required to sustain regular stereocilium architecture and function and that autophagy is pivotal in kinocilium degeneration.

OHC somatic electromotility emerges at P7 in basal-turn OHCs, and at P12 almost all OHCs show motile responses. The response amplitude continues to increase until P13–P14, when mature amplitudes are reached, and this occurs prior to the development of fine tuning^23,24^. The reversible contraction (depolarization)/elongation (hyperpolarization) conformational change in response to the lateral membrane potential takes place thousands of times per second and is mediated by prestin (Slc26a5), and this forms the basis of mammalian cochlear amplification^25–27^. Because OHC somatic motility, but not hair bundle motility, is the basis of the cochlear amplifier, and because somatic motility is another major source contributing to DPOAE, we further sought to determine the OHC electromechanical transduction status upon Atg7 conditional knockout^28^. We evaluated the voltage-dependent charge movement by NLC as a suitable surrogate^29^. As early as P30, typical Atg7-deficient OHCs exhibited reduced NLC/*C*_lin_ and *Q*_max_/*C*_lin_, a *V*_h_ shifted toward a more positive potential, and an increased *z*-value. There was an obvious selection bias of the recordings because (1) the high dynamic range covered up the differences, (2) NLC was recorded in isolated OHCs instead of in situ, and (3) some OHCs failed to be patched and recorded due to dysfunctional membrane status, and thus many of them could have been knockouts. Thus it is possible that there was higher divergence between the two groups than what we have reported.

Another intriguing question was whether Atg7 ablation interfered with OHC synapses because autophagy has been reported to interact with synaptic plasticity, including structural changes in the synapse number, shape, size, and composition^30,31^. After P6, OHCs possess consolidated afferent synapses^32^. Synaptic ribbons of OHCs, although not at every afferent contact, release glutamate in order to weakly and sparsely depolarize unmyelinated type II afferents to convey input^33^. Summation of the excitatory postsynaptic potential from at least six OHCs is required to reach the threshold in type II afferents responding to high-intensity sounds^34^. Our imaging experiments showed that genetic ablation of Atg7 in OHCs provoked rapidly progressing synaptopathy. At P30, the average CtBP2 puncta per OHC was dramatically reduced, especially within the middle/basal turns. Irreversible loss of presynaptic ribbons preceded the loss of OHC cell bodies, and the lack of synaptic input could have contributed to the overall permanent ABR threshold elevation across frequencies. Type II afferents have been demonstrated to be strongly activated by exogenous ATP released from the supporting cells surrounding noise-damaged OHCs^35–37^; however, these afferents were hardly identified in the TEM sections of the knockouts. Together our data suggest that autophagy is indispensable for OHC synaptic signaling.

### Atg7-dependent autophagy/mitophagy is required for OHC mitochondrial homeostasis and cannot be compensated for by other machineries

Bulk autophagy is a non-selective process of degradation of cellular constituents, while mitophagy specifically selects and removes mitochondria^38,39^. Mitochondria provide OHCs with energy production and metabolite synthesis and maintain Ca^2+^ homeostasis. Mitochondria produce cellular ATP via oxidative phosphorylation, supporting the high-energy demands of the cells. However, in the process of producing energy they produce toxic reactive oxygen species^40^. OHCs have evolved a repertoire of intricate quantity and quality control mechanisms to repair or repurpose damaged mitochondria, and selective quarantining and elimination of primed mitochondria through autophagy reduces the release of pro-apoptotic factors into the cytosol that might otherwise activate detrimental downstream pathways^41^. Alterations in mitochondrial function and dynamics can have a negative effect on OHC fitness.

Both in vitro and in vivo evidence supports an active role for autophagy in maintaining the proper action of cochlear HCs and the selective mitochondria clearance process of mitophagy functions in mitochondrial quality and quantity control, thus maintaining a healthy and contextually appropriate mitochondrial population in cochlear HCs^42^. Deficient autophagy/mitophagy can lead to imbalanced Ca^2+^ levels, reduced ATP supply, and subsequent bioenergetics failure^43^. Normal OHC function relies heavily on normal Ca^2+^ and ATP physiology^44^, and the cytoplasmic Ca^2+^ concentration regulates several fast events in OHCs, including the adaptation of MET channels and the release of neurotransmitters at the synapse. Massive numbers of damaged mitochondria inevitably disrupt Ca^2+^ balance and deprive the OHC of its ATP supply^45–49^. Although OHC electromotility does not rely on cellular stores of ATP or Ca^2+^ influx, the proteins involved in the maintenance of transmembrane ion gradients—such as Ca-ATPase, ATP-gated P2X channels, and many others—require an efficient ATP supply^43,50–52^.

The mitophagic machinery has been reported to execute in at least three distinct but interconnected signaling cascades: (1) the ubiquitin-dependent pathway, including foremost the Pink1-dependent Parkin-(in)dependent mitophagy pathway; (2) the receptor-mediated ubiquitin-independent pathway, including Nix, Bnip3, BCL2L13, Fundc1, FKBP8, and others; and (3) the MDV pathway^53–56^. The MDV pathway was specifically involved in aging OHCs, but in the OHCs of Prestin^Cre/+^Atg7^flox/flox^ mice nonselective autophagic vacuoles or MDVs were not found in the Atg7-deficient OHCs at P30, P60, or P90. Robust mitochondria no longer had regularly arranged mitochondrial cristae nor did they have abundant mitochondrial mass. They had moved from their original locations and accumulated, and some of them were partially enwrapped by the limiting membranes. An explanation for these sequestered membranous structures is that because Atg7 plays a fundamental role in the phagophore elongation process the autophagic/mitophagic vacuoles failed to form. Strikingly, there were infrequent mitophagic vacuoles in the degenerating Atg7-deficient OHCs, and these might represent alternative mitochondria digestion pathways (such as Rab9-dependent autophagy) that were induced to counteract mitochondrial deterioration in the absence of Atg7-dependent conventional autophagy/mitophagy machinery, or more likely, these occasional mitophagic vacuoles originated from canonical macroautophagy bypass, thus conferring mechanistic richness^57–59^. However, this compensatory effect was extraordinarily weak and was unable to rescue the OHCs.

**Fig. 7:**
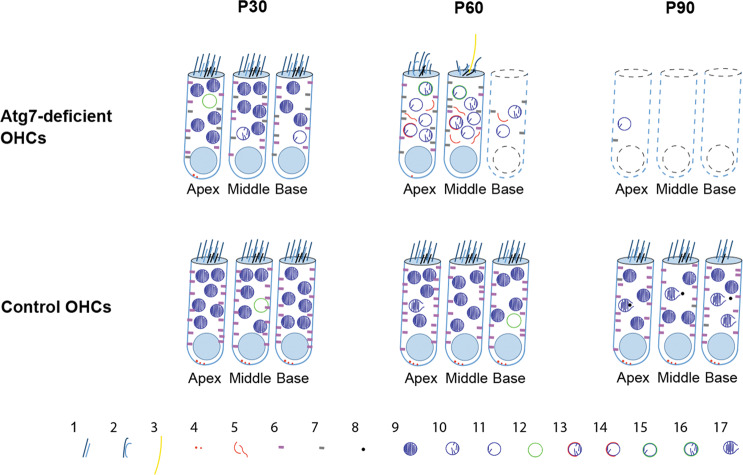
Schematic view of the degeneration of Atg7-knockout OHCs. The upper panel shows the accelerated degeneration of Atg7-deficient OHCs. The lower panel shows control OHCs. Symbols are numbered as follows: (1) stereocilia; (2) disarrayed stereocilia; (3) kinocilium; (4) presynaptic ribbons; (5) sequestering membranes; (6) functional prestin; (7) malfunctional prestin; (8) MDV; (9) mitochondria; (10 and 11) abnormal mitochondria; (12) autophagic vacuoles; (13 and 14) partially enwrapped mitochondria; (15 and 16) fully enwrapped mitochondria; (17) aging mitochondria.

In summary, in the present study we show that disrupted autophagy/mitophagy hampered mitochondrial quality surveillance in OHCs leading to a profound hearing loss phenotype. A significant proportion of the defective mitochondria were only partially engulfed. The activation of Atg7-independent autophagy/mitophagy pathways was insufficient to prevent OHCs from physical deterioration, and susceptible OHCs were finally forced off the stage and ultimately undergo degradation. A schematic view of the phenotype of Atg7-knockout OHCs and the underlying mechanism is proposed in Fig. 7. Collectively, our observations strongly suggest the absolute requirement of Atg7-dependent autophagy/mitophagy for a fully functional mature OHC.

## Conclusion

The malfunction and degeneration of Atg7-deficient OHCs suggested a pronounced sensitivity to autophagy/mitophagy deficiency. Autophagy and mitophagy are highly active processes during inner ear development and maintenance, and these processes quickly dispose of damaged mitochondria. Our results show that the Atg7-dependent autophagy machinery plays a critical role in preserving OHC function and maintaining hearing ability. To what extent autophagy and mitophagy converge and coordinate the regulation of mitochondrial biogenesis and disposal to ensure OHC survival has yet to be fully elucidated in a broader context. Advanced fluorescent reporter systems, such as mito-Timer, mito-QC, mt-Keima, and, most recently, mito-SARI, will hopefully lead to a better understanding of mitophagy both in vitro and in vivo in future studies. In addition, future molecular and genetic studies should be fruitful in unraveling the mystery of the reciprocal interplay between the autophagy and mitophagy pathways in degenerating or injured OHCs and in exploiting for state-of-the-art OHC protection, telling stories of hope.
